# Near-to-patient-testing to inform targeted antibiotic use for sexually transmitted infections in a public sexual health clinic: the NEPTUNE cohort study

**DOI:** 10.1016/j.lanwpc.2023.101005

**Published:** 2024-01-12

**Authors:** Lenka A. Vodstrcil, Kay Htaik, Erica L. Plummer, Vesna De Petra, Melodi G. Sen, Deborah A. Williamson, Jason J. Ong, Jason Wu, Monica Owlad, Gerald Murray, Eric P.F. Chow, Christopher K. Fairley, Catriona S. Bradshaw

**Affiliations:** a Central Clinical School, Monash University, Melbourne, Victoria, Australia; b Melbourne Sexual Health Centre, Alfred Health, Melbourne, Victoria, Australia; c Centre for Epidemiology and Biostatistics, Melbourne School of Population and Global Health, The University of Melbourne, Melbourne, Victoria, Australia; d Microbiological Diagnostic Unit Public Health Laboratory, Department of Microbiology and Immunology, The Peter Doherty Institute for Infection and Immunity at the University of Melbourne, Melbourne, Victoria, Australia; e Department of Microbiology, Royal Melbourne Hospital, Melbourne, Australia; f Victorian Infectious Diseases Reference Laboratory, The Peter Doherty Institute for Infection and Immunity, Melbourne, Victoria, Australia; g Murdoch Children's Research Institute, Parkville, Victoria, Australia; h Women's Centre for Infectious Diseases, The Royal Women's Hospital, Parkville, Victoria, Australia; i Department of Obstetrics and Gynaecology, The University of Melbourne, Parkville, Victoria, Australia

**Keywords:** Near to patient testing, Point of care testing, Mycoplasma genitalium, *Chlamydia trachomatis*, Neisseria gonorrhoeae, Antimicrobial stewardship, Syndromic management, Empiric treatment, Sexually transmitted infections

## Abstract

**Background:**

Empiric treatment of sexually transmitted infections can cause unnecessary antibiotic use. We determined if near-to-patient-testing (NPT) for *Neisseria gonorrhoeae*, *Chlamydia trachomatis* and *Mycoplasma genitalium* (MG) improved antibiotic-use for a range of clinical presentations.

**Methods:**

Clients attending with non-gonococcal urethritis (NGU), proctitis, as STI-contacts, or for an MG-test-of-cure (MG-TOC) between March and December 2021 were recruited. Participants received near-to-patient-testing (NPT-group) for the three STIs using the GeneXpert® System (Cepheid), and concurrent routine-testing by transcription-mediated-amplification (TMA; Aptima, Hologic). Antibiotic-use among NGU or proctitis cases in the NPT-group was compared to clinic-controls undergoing routine-testing only. The proportion in the NPT-group who notified partners <24 hrs of their STI-specific result was calculated.

**Findings:**

Among 904 consults by 808 NPT-participants, ≥1 STI was detected in 63/252 (25.0%) with NGU, 22/51 (43.1%) with proctitis, and 167/527 (31.7%) STI-contacts. MG was detected among 35/157 (22.3%) MG-TOC consults. Among NGU and proctitis cases, fewer in the NPT-group received empiric treatment compared to clinic-controls (29.4% [95% CI: 24.3–34.9%] *vs* 83.8% [95% CI: 79.2–87.8%], p < 0.001), resulting in more NPT-group cases appropriately treated (STI-specific drug/no drug appropriately; 80.9% [95% CI: 76.0–85.1%] *vs* 33.0% [95% CI: 27.7–38.6%], p < 0.001) and fewer mistreated (incorrect drug/treated but pathogen-negative; 17.8% [13.7–22.6%] *vs* 61.4% [55.6–66.9%], p < 0.001). Of 167/264 in the NPT-group with an STI who responded regarding partner-notification, 95.2% notified all/some partners; 85.9% notified them <24 hrs of the STI-specific result.

**Interpretation:**

Near-to-patient-testing significantly improved antibiotic use and a high proportion of individuals rapidly notified partners of STI-specific results, highlighting the broad benefits of timely diagnostic strategies for STIs in clinical decision making and partner notification.

**Funding:**

ARC ITRP Hub-grant; 10.13039/501100000925NHMRC.

**Research in context**

**Evidence before this study**

There has been a rise in STI notifications globally and in Australia, accompanied by increasing rates of antimicrobial resistance for some STIs including *Neisseria gonorrhoeae* and *Mycoplasma genitalium*. Many countries (including Australia) recommend empiric treatment of patients attending with symptoms consistent with an STI, but this can lead to unnecessary antibiotic use. We searched PubMed on July 1, 2023, using the terms (“near to patient testing” OR “point of care” OR “rapid diagnostic”) AND (“*Chlamydia trachomatis*” OR “*Neisseria gonorrhoeae*” OR “*Mycoplasma genitalium*”) NOT (“Review”). Of the 272 identified studies, the majority described assay development, validation and evaluation, prevalence or testing rates of the different infections, and attitudes and acceptability of near-to-patient or point-of-care-testing. Eight included an assessment of the effect of near-to-patient-testing (NPT) on antibiotic use, of which three evaluated antibiotic use following rapid-testing for chlamydia and gonorrhoea in an emergency department compared to a control group. We did not identify any studies examining the impact of NPT on the management or clinical impact of all three bacterial STIs (*C. trachomatis, N. gonorrhoeae, M. genitalium* plus macrolide-resistance-mutation [MRM] testing), or on the following four clinical presentations: non-gonococcal urethritis (NGU), proctitis, STI-contacts and/or clients attending for an *M. genitalium* test-of-cure (MG-TOC).

**Added value of this study**

To the best of our knowledge, this is the largest study to perform NPT for all three bacterial STIs and the first that includes near-to-patient detection of *M. genitalium* MRMs to inform first-line treatment. We assessed the impact of NPT on antibiotic use and partner notification among people presenting with NGU, proctitis, as an STI-contact, or attending for an MG-TOC. Additionally, we compared antibiotic prescribing practices among clients undergoing NPT with the STI syndromes of NGU and proctitis to routine clinical practice (clinic-control group), to determine differences in the proportion appropriately treated, mistreated or overtreated. We also calculated the proportion of all infected participants undergoing NPT who notified their partners of the STI-specific result within 24 h.

NPT detected *C. trachomatis*, *N. gonorrhoeae* and/or *M. genitalium* in 20–40% of clients. Among those with NGU or proctitis the provision of NPT reduced the number receiving empiric treatment, and resulted in an increase in the number receiving appropriate treatment (i.e. STI-specific treatment, or no treatment appropriately) compared to the clinic-control group. This corresponded with a significant reduction in cases with NGU or proctitis undergoing NPT being mistreated (i.e. tested but pathogen negative, or pathogen detected but incorrect drug) or overtreated (i.e. prescribed correct drug but also received additional, unnecessary antibiotics) compared to the clinic-control group. Among all clients with NPT who had an STI detected, most indicated that they notified their sexual partner(s) of their specific result within 24 h. We therefore identified a significant benefit of the rapid provision of results on antimicrobial stewardship and STI-specific partner-notification.

**Implications of all the available evidence**

Antimicrobial resistance, particularly among *N. gonorrhoeae* and *M. genitalium,* is likely to continue rise globally, particularly in the Western-Pacific region where resistance levels are already concerningly high. There have been very few studies examining the impact of NPT as a strategy to improve antimicrobial prescribing practices, and these studies did not include *M. genitalium* or a range of clinical presentations. Our study demonstrates the benefits of NPT for the common STI-syndromes of NGU and proctitis, for STI-contacts and for patients attending for a test-of-cure following treatment of *M. genitalium*. NPT is a strategy that can reduce the time to the delivery of STI-specific treatment and results to patients and their partners and improve antimicrobial stewardship.

## Introduction

Clients attending health services with symptoms or as sexual contacts of individuals with a sexually transmitted infection (STI) commonly receive syndromic treatment with antibiotics in accordance with guidelines.^1^^,^^2^ The use of empiric antibiotics for a syndrome without knowing the infectious cause is also termed presumptive therapy. Empiric treatment aims to cover the main spectrum of aetiologic agents, but as it is not STI-specific, it can lead to antibiotic overuse and misuse. A scoping review of United States-based studies found that only 25–46% patients with symptoms or who were contacts of *Chlamydia trachomatis* (CT) or *Neisseria gonorrhoeae* (NG) had these infections detected.^3^ High levels of antibiotic consumption in countries or certain populations have been associated with increased markers (genotypic and phenotypic) of antimicrobial resistance in STIs in ecological studies.^4^, ^5^, ^6^ The downstream detrimental effects of broad-spectrum antibiotic treatment can extend to other commensal and pathogenic bacteria and bacteria in other anatomical sites (i.e. gut) that were not the target of the treatment. Near-to-patient-testing (NPT; also known as point-of-care testing) refers to diagnostic testing at the time and place of patient care. NPT has the potential to reduce the time to pathogen-specific results and enable clinicians to avoid syndromic treatment and provide treatment for the specific aetiologic agent, thus promoting antimicrobial stewardship. This practice has broad benefits but is likely to be particularly beneficial for STIs prone to antimicrobial resistance, such as NG and *Mycoplasma genitalium* (MG).^7^

Clinical practice at the Melbourne Sexual Health Centre (MSHC) aligns with Australian^1^ and most global guidelines, with empiric prescribing for clients presenting with STI syndromes. STI results are available within 2–5 business days, after which clients are often recalled for STI-specific antibiotics. For clients presenting with the commonest STI syndrome, non-gonococcal urethritis (NGU), empiric treatment on the day of testing is with doxycycline 100 mg *per oral* (PO), twice daily (BD) for 7 days. This therapy effectively treats CT infection and has been shown to reduce the organism load of MG, prior to initiation of resistance-guided therapy with a second targeted antibiotic.^8^ For clients presenting with rectal symptoms consistent with proctitis, empiric treatment involves doxycycline 100 mg PO, BD for 7 d, ceftriaxone 500 mg Intramuscular Injection (IMI) stat and valaciclovir 500 mg PO, BD for 7–10 d to cover CT, NG and herpes simplex virus. The dominant practice for asymptomatic contacts of bacterial STIs globally has been empiric treatment on the day of presentation. This is based on the view that it is beneficial to treat clients immediately in case they do not return, reducing risk of sequelae and ongoing transmission. This practice means asymptomatic contacts commonly receive antibiotics in the absence of an STI.^9^, ^10^, ^11^ Until recently, this was also MSHC practice. However, increasing concerns about antibiotic overuse resulted in MSHC clinicians generally waiting for results and only treating detected infections in contacts, which requires patients to return when laboratory results are available.

In 2021, we examined the impact of NPT for NG, CT and/or MG (plus macrolide-resistance-mutation, MRM) and rapid STI-specific result provision on antibiotic use and partner-notification among clients presenting at MSHC with symptoms consistent with NGU or proctitis, as a sexual contact of an STI, or re-attending for a MG test-of-cure (MG-TOC) following prior treatment (NPT-group). We aimed to compare antibiotic use among NGU or proctitis cases in the NPT-group with use among controls attending for routine-testing (clinic-controls). We also aimed to evaluate the proportion of the NPT-group with an STI-detected who notified their sexual partner(s) within 24 hrs of the STI-specific result.

## Methods

### Study setting and participants

This study was conducted at MSHC, which is the only public sexual health service for ∼5 million people in Melbourne, Australia, with ethics approval from the Alfred Hospital Ethics Committee (ID:369/20). From 18-March to 22-December 2021, the Near-to-patient-testing (NEPTUNE) study recruited clients attending for one of the following reasons: i) symptoms of NGU or proctitis, ii) a sexual contact of CT/NG/MG, or iii) for an MG test-of-cure (MG-TOC) ≥14 days after prior treatment. The triage nurse offered NPT through the NEPTUNE-study and interested, eligible clients were recruited directly by a research team member, or through clinician referral. Clients who declined or were ineligible underwent routine clinical care.

Eligible clients were i) aged 18 years and above, ii) able to provide written informed consent; iii) attended for a reason outlined above; and iv) willing/able to remain in or return to the clinic that day for an appointment to receive treatment and further assessment if indicated. Clients were ineligible if i) the clinician deemed that it was inappropriate to delay assessment (i.e. marked rectal discomfort); ii) they had urethritis with clinical or microscopic signs of NG; or iii) they had additional symptoms. All eligible patients provided written informed consent and completed a short questionnaire to collect data on symptoms and sexual practices. Participants receiving NPT were eligible for NPT a second time if they re-attended the service >8 weeks after their prior visit with new symptoms/a different indication. The specimens collected for NPT were tailored to the reason for attendance; a rectal-swab (proctitis), urine or urethral swab (NGU), or all relevant genital and extra-genital sites (STI-contact/MG-TOC). The NPT-group also received routine laboratory tests outlined below. Specimens were clinician-collected or self-collected using detailed instructions.

### Near-to-patient and standard-of-care routine laboratory

NPT was performed by onsite laboratory staff using the Xpert® CT/NG test (Cepheid, Sunnyvale, CA, US), with CT/NG results in ∼2 hrs. MG and associated MRM testing was with the ***Resistance****Plus*® MG *FleXible* assay (SpeeDx Pty Ltd, Sydney, NSW, Australia), adapted for use with the GeneXpert *FleXible* cartridge format, with results in ∼2.5 hrs.

The standard-of-care laboratory transcription-mediated amplification (TMA) assays used were the Aptima® Combo-2 assay for CT and NG (Hologic Panther system; Hologic, San Diego, CA, US) and the Aptima® MG assay (Hologic). Remnant samples positive for MG were then tested with the ***Resistance****Plus*® MG assay (SpeeDx) for associated MRMs. Performance characteristics of the two assays were also evaluated (see Supplementary Material). Samples that returned an invalid result for a specific test were excluded from the respective analysis and received additional re-testing outside of the study.

The NPT-group were informed that their initial results would be available within 2–3 hrs, and if negative, they would receive an SMS. This SMS reminded the participant to wait for notification of all results from concurrent routine laboratory testing (using assays as above), and HIV and syphilis serology prior to resumption of condomless sex. Participants with an STI detected on NPT were telephoned and asked to return to clinic for treatment as soon as possible, which was predominantly on the same day. MSHC provides free testing and medication, and participants received a pathogen-specific script that was dispensed at the MSHC pharmacy. Participants whose routine laboratory results differed from the NPT results were recalled for further treatment, if required.

### Impact of near-to-patient-testing on antimicrobial stewardship

The research team documented if participants in the NPT-group either i) requested and received syndromic treatment to take and did not wish to wait for results, ii) received a prescription for antibiotics that was only to be dispensed if they received a relevant STI result, or iii) did not receive a prescription and waited for results to inform antibiotic choice.

To estimate the impact of NPT on antimicrobial use, we compared the antibiotic prescribing practices among the NPT-group to a “clinic-control” group. The control group were randomly selected from all clients attending MSHC during the recruitment period who were not in the NPT-group (N = 2481 consultations where a diagnosis of NGU was recorded and N = 564 consultations where a diagnosis of proctitis was recorded), and matched 1:1 with the NPT-group on month of visit, diagnosis code (i.e. proctitis or NGU) and sexual practices (i.e. sex with men or women only) using STATAv17 (StataCorp LP, College Station, TX, USA). Electronic records of controls were extracted and audited by KH, and LG for prescribing practices (i.e. if empiric treatment was provided), blinded to any infections detected.

We defined antimicrobial prescribing as “appropriate” if the client was prescribed the correct drug (recommended by our clinical guidelines) for the specific infection and no additional antibiotics prescribed (e.g. only doxycycline provided and CT detected), or no drug if no infection was detected. “Mistreatment” was defined as the incorrect drug was prescribed for the specific infection (e.g. CT detected but only ceftriaxone provided) or drugs were prescribed but no infection was detected (e.g. empiric treatment but no infection detected). “Overtreatment” was defined as the patient was prescribed the correct drug for the specific infection but also received additional unnecessary antibiotics (e.g. CT detected and patient received doxycycline *and* ceftriaxone).

### Notification of results to sexual partners

Participants in the NPT-group with an STI detected were sent an SMS 24 hrs post-visit with a link to a partner notification survey. This survey asked how many sexual contacts they thought that they should notify, then asked them if they had notified none, some, or all these sexual contacts, and if some or all were notified, approximately what time they notified them.

### Sample size

The sample size required to assess the impact of NPT on antimicrobial prescribing, timely and specific notification of sexual partners, and time to results and appropriate treatment was based on sufficient power to detect a reduction in empiric antibiotic use across all groups and provided precision around each estimate.

First, we estimated that NPT across the two STI syndromes (NGU/proctitis), STI-contacts and MG-TOCs would be associated with **at least** a 30% reduction in empiric antibiotic use. Proctitis is the least common syndrome at MSHC, and we estimated that with 50 NPT-group cases and 50 clinic-controls, we would have >90% power to detect at least a 30% reduction in empiric treatment, from 90% empiric antibiotic use in proctitis-controls compared to 63% in the NPT-group (providing 95% confidence intervals [CIs; precision] around 30% of 17.9–44.6%).

As we planned to recruit greater numbers to each of the other three groups of participants, we had sufficient power to detect at least a 30% reduction in empiric antibiotic use in each group. In addition, we have provided precision (95% CIs) for these estimates. Recruitment of 200 NPT-group participants with NGU provided 95% CIs around 30% of 23.7–36.9%, of 400 STI-contacts provided a 95% CI around 30% of 25.5–34.8%, and 150 MG-TOCs provided a 95% CI around 30% of 22.8–38.0%.

### Statistical analysis

The proportion of participants with each of the STIs detected by NPT with 95% CIs were generated using exact binomial methods (STATA). The two-sample test of proportions was used to compare the proportion of NPT-group participants *vs* clinic-controls who received syndromic treatment, “appropriate treatment”, “mistreatment”, or “overtreatment”, with significance at the level of p < 0.05. Study data were collected and managed using REDCap electronic data capture tools hosted and managed by Helix (Monash University).^12^

### Role of funding source

The funders of the study had no role in the design or conduct of the study, including data collection, management, analysis, or interpretation of the results; preparation, review, or approval of the manuscript; or the decision to submit the manuscript for publication.

## Results

**Fig. 1:**
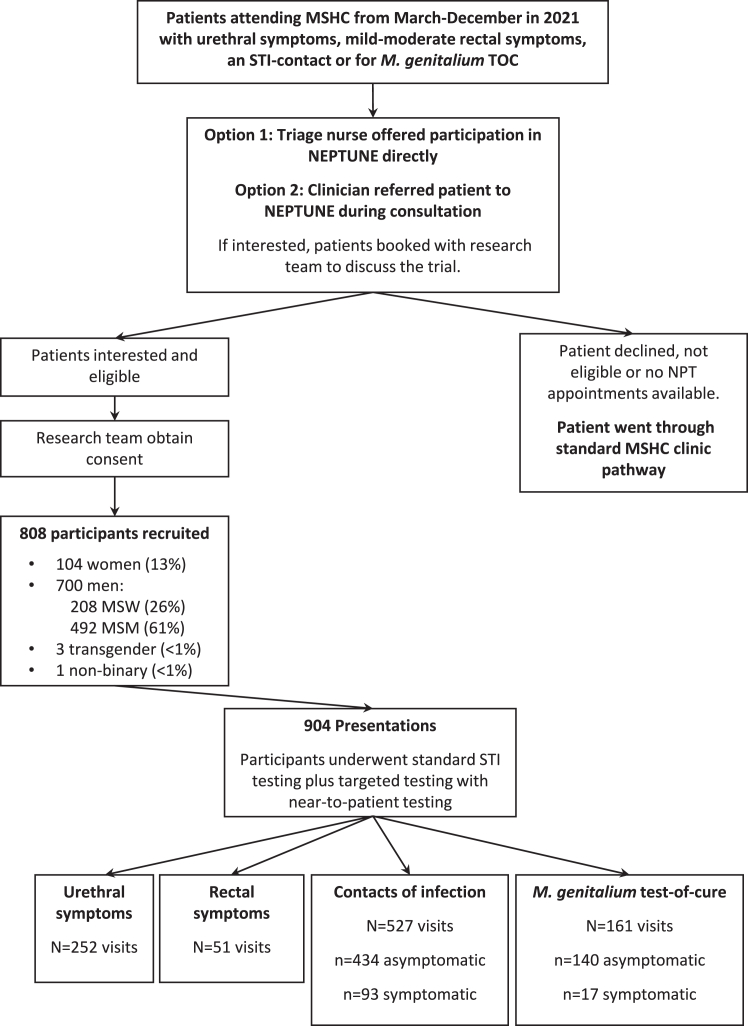
Participant flow chart outlining recruitment to NEPTUNE. Key: MSHC, Melbourne Sexual Health Centre; MSM, men who have sex with men; MSW; men who have sex with women exclusively; NPT, near-to-patient-testing; TOC, test-of-cure.

There were 808 individuals recruited to NEPTUNE for NPT (Fig. 1, Table 1). This included 104 women, 700 men (of which 208 reported sex exclusively with women [MSW] and 492 men who reported sex with men [MSM]), three transgender women and one person who identified as non-binary. Participants were eligible for NPT again if they re-attended the service as defined above; this resulted in a total of 904 NPT-group consultations (Fig. 1, Table 1). For the analysis of antimicrobial prescribing among patients attending with NGU or proctitis, we randomly selected an equivalent number (N = 303) clinic-controls over the study period, matched on STI-syndrome, month of visit and sex or gender of sexual partners.

**Table 1:** Characteristics of the population undergoing near-to-patient-testing (N = 808 individual NPT-participants eligible for at least one analysis).

	Women	Men who have exclusive sex with women	Men who have sex with men	Transgender and non-binary people
	N = 104; 126 consults	N = 208; 229 consults	N = 492; 545 consults	N = 4; 4 consults
	n, %	n, %	n, %	n, %
Age (median [IQR]); individual data only	25 [23–30]	29 [26–34]	32 [28–39]	26 [21–37]
Partner status				
No. male partners last 3 mo (median [IQR])	1 [1–3], range = 0–12	–	4 [2–6], range = 1–150	4 [2–6], range = 0–7
No. female partners last 3 mo (median [IQR])	0 [0–0], range = 0–10	2 [1–3], range = 0–20	0 [0–0], range = 0–7	0 [0–1], range = 0–1
Condom use with male partners				
Always or N/A	32, 25.4	–	69, 12.7	2, 50.0
Sometimes or Never	94, 74.6	–	473, 87.3	2, 50.0
*Missing*	0		3	0
Condom use with female partners				
Always or N/A	116, 92.1	48, 21.0	510, 94.4	4, 100.0
Sometimes or never	10, 7.9	181, 79.0	30, 5.6	0
*Missing*	0	0	5	0
Symptomatic presentation				
Asymptomatic	107, 84.9	142, 62.0	312, 57.3	2, 50.0
Symptomatic	19, 15.1	87, 38.0	233, 42.7	2, 50.0
*M. genitalium* TOC				
No	66, 52.4	171, 75.7	485, 91.3	3, 75.0
Yes	61, 47.6	55, 24.3	46, 8.7	1, 25.0
*Missing*	0	3	14	0
STI-contact				
No	52, 41.3	115, 50.2	199, 36.5	3, 75.0
Yes—*C. trachomatis*	31, 24.6	54, 23.6	154, 28.3	1, 25.0
Yes—*N. gonorrhoeae*	8, 6.35	12, 5.2	106, 19.5	0
Yes—*M. genitalium*	34, 27.0	45, 19.7	43, 7.9	0
Yes—unsure or other	0	1, 0.4	5, 0.9	0
Yes—contact of >1 infection	1, 0.8	2, 0.9	38, 7.0	0

Key: IQR, interquartile range; mo, months; TOC, test-of-cure.

The median age of NPT-group participants with symptoms consistent with proctitis or NGU was similar to randomly selected matched clinic-controls (Table 2). The median number of male or female partners in the last 3 months in the NPT-group was also comparable to clinic-controls (Table 2).

**Table 2:** Infections detected by near-to-patient-testing and/or routine testing among clients with non-gonococcal urethritis or proctitis in the NPT-group or clinic-control group.

Testing group	Non-gonococcal urethritis				Proctitis	
	MSM		MSW		MSM	
	NPT	Clinic	NPT	Clinic	NPT	Clinic
	N = 167 consults; 147 individuals	N = 167 consults; 167 individuals	N = 85 consults; 81 individuals	N = 85 consults; 85 individuals	N = 51 consults; 51 individuals	N = 51 consults; 51 individuals
	n, %	n, %	n, %	n, %	n, %	n, %
Age at consult^a^ (median [IQR])	32 [27–38]	31 [27–38]	31 [26–38]	32 [28–39]	34 [29–42]	30 [25–38]
No. male partners last 3 mo^a^ (median [IQR])	3 [2–6]	3 [2–7]	2 [4–7]	3 [1–5]	–	–
No. female partners last 3 mo^a^ (median [IQR])	0 [0–0]	0 [0–0]	0 [0–0]	0 [0–0]	2 [1–3]	2 [1–3]
*Site of test/detection*	*Urethral*	*Urethral*	*Urethral*	*Urethral*	*Rectal*	*Rectal*
***Near-to-patient-testing—GeneXpert***						
*Total no. bacterial infections detected*						
None	125, 74.9		64, 75.3		29, 56.9	
Monoinfection	42, 25.1		18, 21.2		18, 35.3	
Coinfection	0		3, 3.5		4, 7.8	
*C. trachomatis*						
Not detected	149, 89.2		72, 84.7		46, 90.2	
Detected	18, 10.8		13, 15.3		5, 9.8	
*Invalid*	*0*		*0*		*0*	
*N. gonorrhoeae*						
Not detected	162, 97.0		83, 97.7		40, 78.4	
Detected	5, 3.0		2, 2.3		11, 21.6	
*Invalid*	*0*		*0*		*0*	
*M. genitalium*						
Not detected	144, 88.3		72, 88.9		43, 87.8	
Detected: MRM not detected	4, 2.5		3, 3.7		3, 6.1	
Detected: MRM detected	15, 9.2		6, 7.4		3, 6.1	
*Invalid*	*4*		*4*		*2*	
***Routine testing–Hologic***						
*Total no. bacterial infections detected*^a^						
None	127, 76.0	129, 77.2	62, 72.9	63, 74.1	33, 64.7	33, 64.7
Monoinfection	40, 24.0	36, 21.6	21, 24.7	22, 25.9	15, 29.4	15, 29.4
Coinfection	0	2, 1.2	2, 2.4	0	3, 5.9	3, 5.9
*C. trachomatis*^a^						
Not detected	151, 90.4	147, 88.0	71, 83.5	76, 89.4	46, 90.2	45, 88.2
Detected	16, 9.6	20, 12.0	14, 16.5	9, 10.6	5, 9.8	6, 11.8
*Invalid/Indeterminate*	*0*	*0*	*0*	*0*	*0*	*0*
*N. gonorrhoeae*^a^						
Not detected	162, 97.0	162, 97.0	83, 97.7	83, 97.7	40, 80.0	37, 72.5
Detected	5, 3.0	5, 3.0	2, 2.3	2, 2.3	10, 20.0	14, 27.5
*Invalid/Indeterminate*	*0*	*0*	*0*	*0*	*1*	*0*
*M. genitalium*^a^						
Not detected	148, 88.6	152, 91.0	76, 89.4	74, 87.1	44, 88.0	3^b^
Detected	19, 11.4	15, 9.0	9, 10.6	11, 12.9	6, 12.0	1^b^
*Invalid/Indeterminate*	*0*	*0*	*0*	*0*	*1*	-
***M. genitalium reflexed onto SpeeDx assay***^c^						
Not detected	3, 16.7	4, 26.7	1, 12.5	2, 18.2	2, 33.3	–
Detected: MRM not detected	1, 5.5	2, 13.3	4, 50.0	3, 27.3	0	–
Detected: MRM detected	14, 77.8	9, 60.0	3, 37.5	6, 54.5	4, 66.7	1/1
*Invalid*	*0*	*0*	*0*	*0*	*0*	–
*Result not available*	*1*	*0*	*1*	*0*	*0*	*0*

Key: MSM, men and transwomen who have sex with men; MSW, men who have sex with women exclusively; MRM, macrolide resistance mutation; NPT, near-to-patient-testing group; Clinic, “clinic-controls” attending for standard-of-care/routine testing; NGU, non-gonococcal urethritis; TOC, test-of-cure.

a The proportion of NGU or proctitis consults where an STI was detected was not statistically different between the NPT-group compared to clinic-controls (p > 0.262).

b Guidelines for proctitis recommend routine testing for *C. trachomatis*, *N. gonorrhoeae* and testing for syphilis and HSV, with subsequent testing for *M. genitalium* if symptoms persist or there is a clinical indication. Consequently, only 5/51 of clinic-controls were tested for *M. genitalium* at their initial presentation with symptoms consistent with proctitis.

c Only specimens that had *M. genitalium* detected using the Hologic assay were reflexed onto the SpeeDx assay for MRM testing. There was no SpeeDx assay result available for a small number of samples due to the limitations with using remnant samples.

### The impact of near-to-patient-testing on clinical management

#### STIs and antibiotic use in clients attending with non-gonococcal urethritis

There were 228 individuals in the NPT-group who attended with symptoms and/or signs of NGU representing 252 consultations, Table 2. Of these consultations, 85 were with 81 MSW and 167 were with 146 MSM. The most common symptom reported was dysuria (71.0%), followed by urethral itch (49.6%) and penile discharge (28.6%).

Within NGU clients in the NPT-group, NPT detected infections in 63/252 consults (25.0% positivity); 60 were monoinfected with one of the three infections and three were coinfected with two STIs. Overall, 31/252 (12.3%) had CT, seven (2.8%) NG and 28 (11.1%) MG, of which seven (2.8%) were MRM-negative and 21 (8.3%) were MRM-positive (Table 2). Among the matched clinic-controls with NGU (N = 252 consultations), the proportion with an STI was not significantly different to the NPT-group (Table 2). Routine-testing detected infections in 60/252 clinic-controls with NGU (23.8% positivity); 58 were infected with one of the three infections and two were coinfected with two STIs. When stratified by pathogen, 29/252 (11.5%) had CT, seven (2.8%) NG and 26 (10.3%) MG, of which an MRM-result was available for 20/26 (15/20 MRM-positive, 5/20 MRM-negative).

We then compared prescribing practices between the NPT-group and the clinic-control group with NGU. Among MSM, empiric treatment was received by 53/167 (31.7%, 95% CI: 24.8–39.4) in the NPT-group compared to 144/167 (86.2%, 95% CI: 80.1–91.1) clinic-controls (p < 0.001), Table 3. Appropriate antibiotic therapy was received by 131/167 (78.4%, 95% CI: 71.4–84.4) in the NPT-group compared to 54/167 (32.3%, 95% CI: 25.3–40.0) clinic-controls (p < 0.001). Mistreatment, defined as incorrect treatment requiring recall or treatment when no pathogen was detected, occurred in 36/167 (21.6%; 95% CI: 15.6–28.6) of the NPT-group compared to 113/167 (67.7%, 95% CI: 60.0–74.7) clinic-controls (p < 0.001).

**Table 3:** Antimicrobial prescribing among men and transwomen who presented with symptoms of non-gonococcal urethritis and proctitis in the near-to-patient-testing group (NPT-group) compared to clinic-controls attending for standard-of-care using routine testing.

Syndrome	Clinic pathway	Received empiric treatment	Treatment definition^b^		
			Appropriate treatment	Mistreatment	Over treatment
		n, % [95% CI]	n, % [95% CI]	n, % [95% CI]	n, % [95% CI]
NGU–MSM	NPT N = 167	53, 31.7 [24.8–39.4]	131, 78.4 [71.4–84.4]	36, 21.6 [15.6–28.6]	0
	Clinic N = 167	144, 86.2 [80.1–91.1]	54, 32.3 [25.3–40.0]	113, 67.7 [60.0–74.7]	0
	**p-value**^a^	<0.001	<0.001	<0.001	–
NGU—MSW	NPT N = 85	19, 22.4 [14.0–32.7]	74, 87.1 [78.0–93.3]	11, 12.9 [6.6–22.0]	0
	Clinic N = 85	63, 74.1 [63.5–83.0]	38, 44.7 [33.9–55.9]	46, 54.6 [43.0–65.0]	1^c^, 1.2 [0.03–6.4]
	**p-value**^a^	<0.001	<0.001	<0.001	–
Proctitis^d^–MSM	NPT N = 51	13, 25.5 [14.3–39.6]	40, 78.4 [64.7–88.7]	7, 13.7 [5.7–26.3]	4, 7.8 [2.2–18.9]
	Clinic N = 51	47, 92.2 [81.1–97.8]	8, 15.7 [7.0–28.6]	28, 54.9 [40.3–68.9]	19, 37.3 [24.1–51.9]
	**p-value**^a^	<0.001	<0.001	<0.001	<0.001
*Pooled data*	NPT N = 303	89, 29.4 [24.3–34.9]	245, 80.9 [76.0–85.1]	54, 17.8 [13.7–22.6]	4, 1.3 [0.4–3.3]
	Clinic N = 303	254, 83.8 [79.2–87.8]	100, 33.0 [27.7–38.6]	186, 61.4 [55.6–66.9]	21, 6.9 [4.3–10.4]
	**p-value**^a^	<0.001	<0.001	<0.001	0.154

Key: MSM, men and transwomen who have sex with men; MSW, men who have sex with women exclusively; NPT, near-to-patient-testing tested with GeneXpert; Clinic, “clinic-controls” attending for standard of care; NGU, non-gonococcal urethritis.

a p-value for two sample test of proportions comparing NPT group *vs* clinic control group.

b Definitions: “Appropriate treatment” appropriate antibiotic, no excess antibiotic, or no antibiotic provided and no pathogen detected; “Mistreatment”: mistreated with incorrect antibiotic requiring recall, or treated in absence of a pathogen; “Over treatment”: over treated with correct antibiotic and additional antibiotic/s.

c One client received ceftriaxone in addition to doxycycline because they were a contact of *N. gonorrhoeae*, but only *C. trachomatis* was detected.

d Guidelines for proctitis recommend routine testing for *C. trachomatis*, *N. gonorrhoeae* and testing for syphilis and HSV, with subsequent testing for *M. genitalium* if symptoms persist or there is a clinical indication. Consequently, only 4/51 of clinic-controls were tested for *M. genitalium* at their initial visit. Regardless, these clients still wouldn't have received the correct treatment. NEPTUNE enabled *M. genitalium* and associated MRM testing at point-of-care so that the result was known within 3 h and appropriate treatment provided at the same time.

Among MSW with NGU, empiric treatment was received by 19/85 (22.4%, 95% CI: 14.0–32.7) in the NPT-group and 63/85 (74.1%, 95% CI: 63.5–83.0) clinic-controls (p < 0.001), Table 3. Appropriate treatment was received by 74/85 (87.1%; 95% CI: 78.0–93.3) in the NPT-group compared to 38/85 (44.7%; 95% CI: 33.9–55.9) clinic-controls (p < 0.001), whereas mistreatment occurred in 11/85 (12.9%; 95% CI: 6.6–22.0) of the NPT-group *vs* 46/85 (54.6%; 95% CI: 43.0–65.0) clinic-controls (p < 0.001).

Among the NPT-group, GeneXpert detected one additional CT infection compared to TMA, but the same number of MG infections (Table 2 and Supplementary Material). Of the positive MG specimens by TMA reflexed for MRM testing; four did not have MG detected, five were MRM-negative and 17 were MRM-positive.

#### STIs and antibiotic use in patients attending with proctitis

Fifty-one people (MSM and transgender women) in the NPT-group had rectal symptoms consistent with proctitis, Table 2. The most common rectal symptoms for the NPT-group were itch (84.3%) and pain (62.8%); 49.0% reported pain with defecation, 39.2% discharge, and 27.5% rectal bleeding.

NPT detected infections in 22/51 with symptoms of proctitis (43.1% positivity); eighteen were infected with one bacterial STI and four were coinfected with two STIs. Overall, among the NPT-group, 5/51 (9.8%) had CT, 11/51 (21.6%) NG and 6/51 (11.8%) MG, of which 3/51 (5.9%) were MRM-negative and 3/51 (5.9%) MRM-positive (Table 2). For the clinic-control group, guidelines for proctitis recommend routine-testing for CT and NG (as well as syphilis and HSV-testing), and only testing for MG if symptoms persist or there is a clinical indication. Consequently, only 4/51 of clinic-controls were tested for MG at their initial presentation with symptoms consistent with proctitis. Among matched clinic-controls with proctitis (N = 51), there was no significant difference in the overall proportion with an STI-detected. Routine-testing detected infections in 18/51 (35.3% positivity) of clinic-controls with proctitis; fifteen were monoinfected and three were coinfected with two STIs. When stratified by pathogen, 6/51 (11.8%) of clinic-controls with proctitis had CT, fourteen (27.5%) NG and one (2.0%) had MG (MRM-positive).

Empiric therapy was received by 13/51 (25.5%; 95% CI: 14.3–39.6) in the NPT-group with proctitis, compared to 47/51 (92.2%; 95% CI: 81.1–97.8) clinic-controls, Table 3. Appropriate antibiotic therapy was given to 40/51 (78.4%; 95% CI: 64.7–88.7) in the NPT-group compared to 8/51 (15.7%; 95% CI: 7.0–28.6) clinic-controls (p < 0.001). Mistreatment occurred in 7/51 (13.7%; 95% CI: 5.7–26.3) in the NPT-group compared to 28/51 (54.9% 95% CI: 40.3–68.9) clinic-controls (p < 0.001), and overtreatment, defined as correct treatment, but additional drug/s, occurred in 4/51 (7.8%; 95% CI: 2.2–18.9) in the NPT-group *vs* 19/51 (37.3%; 95% CI: 24.1–51.9) clinic-controls (p < 0.001).

We also pooled the antimicrobial use data within all participants with a syndrome in the NPT-group and clinic-control group, Table 3. Appropriate treatment was received by 245/303 (80.9%; 95% CI: 76.0–85.1) patients with an STI syndrome in the NPT-group compared to 100/303 (33.0%; 95% CI: 27.7–38.6) clinic-controls (p < 0.001), whereas mistreatment occurred in 54/303 (17.8%; 95% CI: 13.7–22.6) the NPT-group *vs* 186/303 (61.4%; 95% CI: 55.6–66.9) clinic-controls (p < 0.001).

All participants in the NPT-group with proctitis provided a sample/s for concurrent routine testing by TMA. When compared to TMA, GeneXpert detected one additional NG infection and one fewer MG-infection (Table 2). Positive MG specimens by TMA subsequently tested for MRMs using the SpeeDx assay identified four MRM-positive infections; two did not have MG detected. Additional data on the two assays is presented in Supplementary Material.

#### STIs and antibiotic use in patients attending as sexual contacts-of-infection

There were 491 individuals who attended 527 NPT consultations in which the individual reported being a contact of either CT (n = 277 consults), NG (n = 156 consults) or MG (n = 127 consults) or contact of >1 infection (n = 41 consults), Table 4. Individuals were triaged into the clinic as either asymptomatic (434 consultations) or symptomatic (93 consultations) contacts-of-infection.

**Table 4:** Infections detected by near-to-patient-testing and/or routine testing among clients declaring to be a contact-of-infection.

	All contacts-of-infection	Stratified by symptomatic status		Stratified by sex or sexual orientation		
		Asymptomatic^a^	Symptomatic^a^	Female/has vagina	MSW	MSM
	N = 527 consults (491 individuals)	N = 434 consults (405 individuals)	N = 93 consults (86 individuals)	N = 75 consults (68 individuals)	N = 113 consults (107 individuals)	N = 339 consults (316 individuals)
	n, %	n, %	n, %	n, %	n, %	n, %
Age (median [IQR])	30 [26–36]	29 [25–35]	32 [26–37]	25 [22–29]	25 [25–32]	32 [27–38]
Sex and/or sexual orientation						
Women and other people with a vagina	68, 13.8	53, 13.1	15, 17.6			
MSW	107, 21.8	94, 23.2	13, 15.1			
MSM	316, 64.4	258, 63.7	58, 67.4			
Attending for *M. genitalium* TOC						
No	494, 95.9	408, 96.2	86, 94.5	67, 89.3	103, 92.8	324, 98.5
Yes	21, 4.1	16, 3.8	5, 5.5	8, 10.7	8, 7.2	5, 1.5
*Missing data*	*12*	*10*	*2*	*0*	*2*	*10*
STI contact						
Yes—*C. trachomatis*	239, 45.3	203, 46.8	36, 38.7	32, 42.7	54, 47.8	153, 45.1
Yes—*N. gonorrhoeae*	125, 23.7	101, 23.3	24, 25.8	8, 10.7	12, 10.6	105, 31.0
Yes—*M. genitalium*	122, 23.2	95, 21.9	27, 29.0	34, 45.3	45, 39.8	43, 12.7
Yes–contact of >1 infection	41, 7.8	35, 8.1	6, 6.5	1, 1.3	2, 1.8	38, 11.2
Symptomatic presentation						
No	434, 82.3			58, 77.3	100, 88.5	276, 81.4
Yes	93, 17.7			17, 22.7	13, 11.5	63, 15.6
***Same infection detected as declared COI (any site)—Near-to-patient testing–GeneXpert***						
*C. trachomatis* COI [N = 277 consults]						
Not detected	199, 72.4	170, 72.3	29, 72.5	26, 78.8	43, 76.8	130, 69.9
Detected	76, 27.6	65, 27.7	11, 27.5	7, 21.2	13, 23.2	56, 30.1
*Invalid*	*2*	*2*	*0*	*0*	*0*	*2*
*N. gonorrhoeae* COI [N = 156 consults]						
Not detected	124, 80.5	104, 80.6	20, 80.0	8, 100.0	12, 92.3	104, 78.2
Detected	30, 19.5	25, 19.4	5, 20.0	0	1, 7.7	29, 21.8
*Invalid*	*2*	*2*	*0*	*0*	*0*	*2*
*M. genitalium* COI [N = 127 consults]						
Not detected	89, 71.2	69, 71.1	20, 71.4	26, 74.3	30, 69.8	33, 70.2
Detected–MRM negative	9, 7.2	6, 6.2	3, 10.7	4, 11.4	4, 9.3	1, 2.1
Detected–MRM positive	27, 21.6	22, 22.7	5, 17.9	5, 14.3	9, 20.9	13, 27.7
*Invalid*	*2*	*2*	*0*	*0*	*2*	*0*
***Same infection detected as declared COI (any site)—Routine testing–Hologic***						
*C. trachomatis* COI [N = 277 consults]						
Not detected	197, 72.4	171, 73.1	26, 68.4	26, 78.8	42, 75.0	129, 70.5
Detected	75, 27.6	63, 26.9	12, 31.6	7, 21.2	14, 25.0	54, 29.5
*Invalid*	*5*	*3*	*2*	*0*	*0*	*5*
*N. gonorrhoeae* [N = 156 consults]						
Not detected	123, 80.4	104, 80.6	19, 79.2	7, 87.5	12, 92.3	107, 78.7
Detected	30, 19.6	25, 19.4	5, 20.8	1, 12.5	1, 7.7	29, 21.3
*Invalid*	*3*	*2*	*1*	*0*	*0*	*2*
*M. genitalium* COI [N = 127 consults]						
Not detected	84, 66.1	67, 67.7	17, 60.7	22, 62.9	29, 64.4	33, 70.2
Detected	43, 33.9	32, 32.3	11, 39.3	13, 37.1	16, 35.6	14, 29.8
***M. genitalium reflexed onto SpeeDx assay***^b^						
Not detected	4, 10.5	2, 7.1	2, 20.0	1, 7.7	3, 23.0	0
Detected–MRM negative	13, 34.2	9, 32.1	4, 40.0	6, 46.15	5, 38.5	2, 16.7
Detected–MRM positive	21, 55.3	17, 60.7	4, 40.0	6, 46.15	5, 38.5	10, 83.3
*Invalid*	*1*	*1*	*0*	*0*	*0*	*1*
*Result not available*	*4*	*3*	*1*	*0*	*3*	*1*
***Different infection detected as declared COI (any site)–GeneXpert***						
*C. trachomatis (NG COI)*	***n = 127***	***n = 102***	***n = 25***	***n = 8***	***n = 12***	***n = 107***
Not tested	2	1	1	1	0	1
Not detected	114, 91.9	94, 94.0	20, 83.3	7	9, 75.0	98
Detected^c^	10, 8.1	6, 6.0	4, 16.7	0	3, 25.0	7
*Invalid*	*1*	*1*	*0*	*0*	*0*	*1*
*N. gonorrhoeae (CT COI)*	***n = 248***	***n = 208***	***n = 40***	***n = 33***	***n = 55***	***n = 160***
Not tested	5	5	0	1	4	0
Not detected	223, 92.1	190, 94.1	33, 82.5	29	51	143
Detected	19, 7.9	12, 5.9	7, 17.5	3	0	16
*Invalid*	*1*	*1*	*0*	*0*	*0*	*1*

Key: COI, contact-of-infection; IQR, interquartile range; MSM, men and transwomen who have sex with men; MSW, men who have sex with women exclusively; MRM, macrolide resistance mutation; TOC, test-of-cure.

N.B. For STI-contacts, results reflect positivity for the infection declared to be contact of at any site that was tested. Overall, ≥1 bacterial STI was detected in 167/527 (31.7%) consults using the GeneXpert and in the same participants in 183/527 (34.7%) routine tests with Hologic TMA assays (p=0.301). Coinfections were not tabulated because participants undergoing NPT were not all routinely tested for other infections.

a There was no statistically significant difference in the proportion of symptomatic *vs* asymptomatic contacts-of-infection who had the STI that they declared to be a contact of (p > 0.859).

b Only specimens that had *M. genitalium* detected using the Hologic assay were reflexed onto the SpeeDx assay for MRM testing. There was no SpeeDx assay result available for a small number of samples due to the limitations with using remnant samples, especially for low-load asymptomatic infections.

c A higher proportion of symptomatic than asymptomatic individuals had a different infection detected than the one declared. However, this was only significant for CT-contacts who had *N. gonorrhoeae* detected (p = 0.013).

Among CT-contacts undergoing NPT, 76/275 (27.6%) had CT detected, with no difference between asymptomatic and symptomatic contacts (27.7% *vs* 27.5%, respectively), Table 4. Among NG-contacts, there were 30/154 (19.5%) with NG detected, with no difference when stratified by symptomatic status (19.4% *vs* 20.0%). Finally, among MG-contacts, there were 36/125 (28.8%) MG infections; nine that were MRM-negative and 27 that were MRM-positive, with no difference in the proportion with MG overall when stratified by symptomatic infections (28.9 *vs* 28.6%).

We next calculated the proportion of individuals undergoing NPT who reported to be a contact-of-infection but who had a different infection detected (Table 4). This was only done for participants reporting to be contacts of CT or NG as these individuals were routinely tested for the other infection as part of the same GeneXpert cartridge (as well as by TMA). Of 124 NG-contacts, 10 (8.1%) had CT detected; with three coinfections. Of 242 CT-contacts, 19 (7.9%) had NG detected; with five coinfections. A higher proportion of symptomatic than asymptomatic individuals had a different infection detected than the one declared. However, this was only significant for CT-contacts who had NG (17.5% among symptomatic *vs* 5.9% among asymptomatic patients, p = 0.013).

Of all consults with contacts-of-infection, ≥1 bacterial STI was detected among 167/527 (31.7%) cases tested using GeneXpert and 183/527 (34.7%) consults tested using TMA assays (p = 0.301), Table 4. Compared to GeneXpert results, TMA detected one fewer CT infection among chlamydia contacts, but seven additional MG infections among MG-contacts (Table 4, Supplementary Material). Of these additional MG infections, four were asymptomatic individuals and three symptomatic.

#### *STIs and antibiotic use in clients attending for an* MG-TOC

There were 108 individuals who attended 161 NPT consultations for an MG-TOC, which is routinely recommended following treatment for MG at MSHC, Table 5. Their mean age was 29 (IQR 25–37), over half had a current regular sexual partner (63.1%), and in 16/161 consults, participants reported being symptomatic.

**Table 5:** Infections detected by near-to-patient-testing and/or routine testing among clients attending for an *M. genitalium* test-of-cure.

		Stratified by symptomatic status	
	All MG-TOCs	Asymptomatic^a^	Symptomatic^a^
	N = 108 individuals, 161 visits	N = 96 individuals, 145 visits	N = 12 individuals, 16 visits
	n, %	n, %	n, %
Age (median [IQR])	29 [25–37]	29 [25–36]	27 [25–39]
Sex and/or sexual orientation			
Women and other people with a vagina	61, 37.9	56, 38.6	5, 31.3
MSW	54, 33.5	49, 33.8	5, 31.3
MSM	46, 28.6	39, 26.9	7, 43.8
***M. genitalium testing***			
***Near-to-patient-testing GeneXpert***			
Not detected	122, 77.7	108, 77.1	14, 82.4
Detected–MRM negative	5, 3.2	5, 3.6	0
Detected–MRM positive	30, 19.1	27, 19.3	3, 17.7
*Invalid*	*4*	*4*	*0*
***Routine testing Hologic***			
Not detected	112, 70.0	100, 69.9	12, 70.6
Detected	48, 30.0	43, 30.1	5, 29.4
*Invalid*	*1*	*1*	*0*
***M. genitalium reflexed onto SpeeDx assay***^b^			
Not detected	10, 24.4	10, 26.3	0
Detected–MRM negative	4, 9.8	4, 10.5	0
Detected–MRM positive	27, 65.8	24, 63.2	3, 100.0
*Invalid*	*1*	*1*	*0*
*Result not available*	*6*	*4*	*2*

Key: MSM, men and transwomen who have sex with men; MSW, men who have sex with women exclusively; MRM, macrolide resistance mutation.

a There was no statistically significant difference in the proportion of symptomatic *vs* asymptomatic participants attending for an MG-TOC (p > 0.760).

b Only specimens that had *M. genitalium* detected using the Hologic assay were reflexed onto the SpeeDx assay for MRM testing. There was no SpeeDx assay result available for a small number of samples due to the limitations with using remnant samples, especially for low-load asymptomatic infections.

NPT detected 35/157 (22.3%) MG infections that occurred in 32 asymptomatic patients and 3 symptomatic patients (Table 5); five (3.2%) were MRM-negative and 30 (19.1%) were MRM-positive; four tests were invalid. In comparison, TMA detected 48/160 (30.0%) MG infections; 43 were asymptomatic and five symptomatic. TMA-positive specimens then referred for MRM testing returned a result for MRM status for 31 samples (four MRM-negative and 27 MRM-positive), one was invalid and ten did not detect MG.

### Partner notification data

Overall, of the 264 participants in the NPT-group with an STI detected by NPT (Table 6), 167/264 (63.3%) responded to an SMS sent 24 hrs after notification of their result; of the 167 who responded, 159 (95.2%) reported that they notified some or all sexual partners of the STI-specific result. Over half reported that they notified their partner/s on the same day that they had received their result and a third notified their partner on the subsequent day. Similar data were not available for an equivalent clinic-control group, however current clinic procedures include notifying patients of their STI-specific result on average 2–4 days after presentation. Therefore, NPT-participants had a significantly shortened time-to-result compared to clients undergoing routine testing, and therefore all NPT-participants could rapidly notify their partners of their STI-specific result.

**Table 6:** Partner notification data for 264 participants who had STI detected with near-to-patient-testing.

	Had an STI detected with NPT	Not a contact of infection	Reports to be a sexual contact of infection
	N = 264	n = 97	n = 167
	n, %	n, %	n, %
STI diagnosed with			
*C. trachomatis*	105, 39.8	28, 28.9	77, 46.1
*N. gonorrhoeae*	50, 18.9	10, 10.3	40, 23.9
*M. genitalium*	91, 34.5	53, 54.6	38, 22.8
>1 STI diagnosis	18, 6.8	6, 6.2	12, 7.2
Sex and/or sexual orientation			
Women and other people with a vagina	33, 12.5	14, 14.4	19, 11.3
MSW	55, 20.8	26, 26.8	29, 17.4
MSM	176, 66.7	57, 58.8	119, 71.3
Notified partners (from the last 3 mo)			
Missing/Did not respond to SMS	97, 36.7	39, 40.2	58, 34.7
No	8, 3.0	6, 6.2	2, 1.2
Yes–Some	42, 15.9	10, 10.3	32, 19.2
Yes–All	117, 44.3	42, 43.3	75, 44.9
How many partners (from the last 3 mo) do you need to notify?	*n = 42 responses*	*n = 10 responses*	*n = 32 responses*
Median [IQR], range	2 [2–4], range = 0–13	2 [1–3], range = 1–10	2 [2–5], range 0–13
Days to notification	*n = 164 responses*	*n = 54 responses*	*n = 110 responses*
0 (same day)	91, 55.5	26, 48.1	65, 59.1
1 day	50, 30.5	19, 35.2	31, 28.2
2+ days	23, 14.0	9, 16.7	14, 12.7

Key: IQR, interquartile range; MSM, men and transwomen who have sex with men; MSW, men who have sex with women exclusively; MRM, macrolide resistance mutation mo, months.

## Discussion

The NEPTUNE study aimed to assess the benefits of NPT for patients in a large urban sexual health service presenting with the common STI syndromes of NGU and proctitis, as an STI-contact or for an *M. genitalium* test-of-cure. In two-thirds of the participants who received NPT, no bacterial STI was detected, highlighting the advantage of timely, aetiologic strategies to mitigate antibiotic overuse and misuse in settings where syndromes and contacts are commonly treated empirically. Specifically, NPT identified a pathogen in 25–40% of participants with NGU or proctitis symptoms. Additionally, <30% of participants who stated they were a sexual contact of an individuals with an STI were infected with the STI they reported exposure to. Our assessment of antibiotic prescribing found that participants in the NPT-group were significantly more likely to get appropriate treatment (i.e. correct drug or no drug/no excess drug) (81% *vs* 24%) and were less likely to be mistreated (17% *vs* 70%) compared to clinic-attendees. Importantly, this study confirms the need to avoid presumptive treatment of contacts of STIs. Finally, most participants in the NPT-group reported rapid rates of STI-specific partner-notification, which demonstrates an additional benefit of expedited result provision.

Our findings demonstrate that rapid and accurate NPT has the potential to enhance STI-control through timely results and partner notification and pathogen-directed treatment. This is in line with the *WHO Global Health Sector Strategy for the control and prevention of STIs*, which outlines the importance of integrating point-of-care tests, diagnosis, and treatment into a single visit.^13^ In our busy, but relatively well-resourced, urban service, NPT and swift notification of results to patients provided clinicians with an option to delay presumptive therapy and deliver aetiologically directed appropriate treatment. Very few published studies have assessed the impact of NPT on clinical care, and of the three that specifically looked antibiotic use, all were conducted at Emergency Departments in the US and none included MG. However, these three studies also found that rapid-testing (using GeneXpert) and delivery of CT and NG results increased the likelihood clients were present when the result was ready and decreased unnecessary antibiotic exposure.^14^, ^15^, ^16^ Although all NPT-participants returned for treatment after 2–3 hrs, the optimal time to result has been specified as within 30–60 min,^17^ with an even greater reduction in time from test to result necessary to ensure the patient does not leave before etiologic treatment.^18^ A more rapid form of the Xpert® CT/NG test is in development, however, the manufacturer does not have a release date at this time. While the ***Resistance****Plus*® MG FleXible assay used was the slowest assay at ∼2.5 hrs, the newer simplified assay still provides a result in the order of 2 hrs. Importantly, one of the main barriers to expanded roll-out of this approach is the upfront cost of point-of-care systems. The WHO states that the minimal acceptable target price per test is <US$5 (excluding the cost of a device or reader) and <US$1 is the optimal price.^17^ A cost-effectiveness analysis of our NPT approach compared to our current clinic practice is underway to determine the distribution of healthcare costs at all stages of service delivery to inform the feasibility of its expanded use in our setting. Additionally, we are evaluating the patient and clinician acceptability of this strategy.

Some of the greatest benefits of NPT on antimicrobial stewardship in our study were seen among patients with symptoms of an STI syndrome. Amongst NPT-participants with suspected NGU, less than 30% had an STI detected, which largely reflected an equal number of CT and MG infections, and a small number of NG cases. Whilst empiric doxycycline for NGU treats CT and is part of the sequenced resistance-guided strategy for MG^8^ in Australian, British and US guidelines, doxycycline was only definitively indicated in 1:4 of these clients (with CT and/or MG detected). NPT significantly reduced empiric therapy in NGU, with fewer NPT-participants receiving an unnecessary drug compared to a clinic-control group, however there is also some evidence that doxycycline may reduce inflammation among NGU cases in the absence of a known pathogen^19^ or may cure idiopathic urethritis so any benefit for such cases still remains to be determined.^20^ Conversely, this practice will result in other organisms being inadvertently exposed to doxycycline, unwanted effects on the human microbiome, and tetracycline-use may also induce resistance in some organisms including NG.^21^^,^^22^

A higher proportion of proctitis cases had an STI detected (41%), with NG the most common infection detected. The higher STI prevalence among patients with proctitis is consistent with other studies of symptomatic men.^23^, ^24^, ^25^ This finding could indicate that more patients would benefit from presumptive treatment. However, current empiric treatment includes three drugs (ceftriaxone, doxycycline and valaciclovir) to provide coverage for the three most common infectious causes of proctitis, so a high proportion of patients are exposed to additional, unrequired drug(s). This is of particular concern when current efforts are being directed towards reducing ceftriaxone use as the last effective drug for NG. Of note among the clinic-control group, 20/51 unnecessarily received ceftriaxone. Overall, there were additional gains achieved with pathogen-directed treatment among NPT-participants, with fewer individuals mistreated and overtreated compared to a clinic-control group.

Fewer than 30% of STI-contacts receiving NPT had an STI detected. This finding aligns with previous studies of MG, CT and NG detection among STI-contacts at MSHC,^9^, ^10^, ^11^^,^^26^ and supports the cessation of empiric treatment of asymptomatic contacts at our service. If STI-contacts receiving NPT had been empirically treated, 70–80% would have received an unnecessary antibiotic. This finding is comparable to studies in similar urban STI clinic populations reporting unnecessary treatment of contacts.^27^^,^^28^ A recent study at the Sydney Sexual Health Centre found that a switch from empiric treatment of all asymptomatic contacts to treating only detected infections significantly reduced antibiotic overuse with minimal adverse outcomes.^28^ Many services are starting to divert from empiric treatment of contacts; however, guidelines vary internationally. Australian guidelines recommend that clinicians “consider empiric treatment if there has been sexual contact within the past 2 weeks or when the person's individual circumstances mean later treatment may not occur”,^1^ while empiric treatment of contacts is recommended under specific circumstances in the US and Europe.^2^^,^^29^

Amongst NPT-participants attending for an MG-TOC review, a third had MG detected, and detection was not associated with symptoms. These data show that absence of symptoms does not mean clearance of infection. Assuming they were infected with a viable organism, this suggests that 1 in 3 treated patients were at risk of unknowingly transmitting MG to others. Overall, MG is a more problematic organism for the majority of NAAT assays to detect, as it tends to occur at lower loads than both CT and NG. This was reflected in our comparison of GeneXpert to TMA (Supplementary Material), where, consistent with published literature,^30^^,^^31^ the GeneXpert system had high sensitivities and specificities for CT and NG compared to TMA. The greatest number of discrepancies were observed between GeneXpert and TMA for MG, reflecting several issues that have been outlined in the Supplementary Material.

### Strengths and limitations

With >800 participants, this was the largest study of NPT at an urban clinic and included NPT for three STIs among participants attending with STI-syndromes, STI-contacts, and for an MG-TOC. This sample size allowed us to measure the effects of NPT for several clinically relevant outcomes as discussed. Of note, the number of partners notified promptly of their exposure to an STI may have been even higher than reported, as 36% of NPT-participants did not respond to the SMS about partner notification. One of the main limitations was that the COVID-19 pandemic disrupted the clinic's workflow and laboratory,^32^ with downstream impacts on study recruitment, staff availability and other priorities in the on-site laboratory. There also may have been other causative pathogens of proctitis and NGU that were not identified that may have benefited from empiric treatment. A randomised controlled trial with extended follow up would increase our understanding of the implications of not using antibiotics in patients with a syndrome who test negative for a bacterial pathogen. Our study did not collect information on contact tracing from controls but as controls were unable to be notified of the STI-specific result <24 hrs, no comparison could be made. For our comparison of syndromic management, we randomly selected clinic-controls diagnosed with NGU and proctitis to match those undergoing NPT, and there were no significant epidemiological and microbiological differences between groups. The only exception was that people with proctitis in the clinic-control group were not all tested for MG at their initial presentation, in line with international recommendations.^1^^,^^2^^,^^33^ If they had been tested for MG and it was detected, most would have been overtreated if treated as per current guidelines (i.e. would have received ceftriaxone, valtrex and doxycycline). In addition, given the large effect sizes we observed it is unlikely that any unmeasured differences between groups affected our findings. Finally, as mentioned above, our cost-effectiveness analysis will be able to determine how cost-effective this approach is at our service for each of the indications.

### Conclusion

Near-to-patient-testing for STIs has considerable potential to improve antibiotic prescribing practices and the downstream detrimental effects on individuals, pathogens and the community. In this study we demonstrated that implementation of NPT in a well-resourced urban setting achieves rapid and accurate diagnosis among people with STI syndromes and STI-contacts and improves the proportion of people receiving appropriate treatment. The result was immediate improvement in antimicrobial use. The additional benefits that flow on from rapid STI-specific partner notification could not be measured in this study but may include sexual partners promptly attending for testing with accurate information regarding the STI they have been exposed to, reducing their risk of sequelae and further transmission. While the success of NPT systems relies on the rapid dissemination of results, accurate point-of-care systems also need to be affordable and accessible for a wide range of healthcare settings to make a broader impact on health outcomes.

## Contributors

LAV performed the data analysis and wrote the first draft of the manuscript. CSB conceived and designed the study, with input from LAV, KH and ELP. KH, MO, and JW were responsible for conducting the participant recruitment and data collection. CSB and KH had access to and verified the data. VDP and MGS were responsible for conducting the laboratory assays, with input from DWW. All authors provided data interpretation, revised the manuscript for intellectual content, and approved the final version of the manuscript. The corresponding authors had full access to all the data in the study and had final responsibility for the decision to submit for publication.

## Data sharing statement

The data that support this study are included in the article. Further inquiries can be directed to the corresponding author and the availability of the data will be subjected to the permission of the Alfred Hospital Ethics Committee (ID:369/20).

## Declaration of interests

This trial was funded by an ARC ITRP Hub Grant Project ID IH190100021. CSB and CSK were supported by an Australian NHMRC Leadership Investigator Grant (GNT1173361 and GNT1172900, respectively). DEW, JJO and EPFC were supported by an NHMRC Emerging Leadership Investigator Grant (GNT1174555, GNT1193955 and GNT1172873, respectively).
