# Supplementary material for: Near-to-patient-testing to inform targeted antibiotic use for sexually transmitted infections in a public sexual health clinic: the NEPTUNE cohort study
Source: Lancet Reg Health West Pac. 2024 Jan 12;44:101005. doi: 10.1016/j.lanwpc.2023.101005 (PMC10825687; doi:10.1016/j.lanwpc.2023.101005)
Supplement: Supplementary Material [file mmc1.docx]

***Supplementary Material: Additional data regarding assay performance***

We determined the positive and negative percent agreement (PPA and NPA, respectively) and Cohen’s kappa agreement (coefficient) for the GeneXpert® System compared to the Hologic TMA assays (Table 1, below), with 95% confidence intervals using STATA. For all values, data from all eligible participants in the near-to-patient-testing group and sampling sites were combined, and only infections returning detected/not-detected results were compared (i.e., invalid results were excluded).

For *N. gonorrhoeae* and *C. trachomatis*, the Xpert® CT/NG test had comparable performance statistics to the Hologic TMA assays (Table 1). The PPA and NPA for *N. gonorrhoeae* were 95·5% (95%CI: 87·3-99·1%) and 99·8% (95%CI: 99·0-100·0%), respectively and for *C. trachomatis* were 94·8% (95%CI: 89·1-98·1%) and 98·9% (95%CI: 97·6-99·6%), respectively. For *M. genitalium*, both the Hologic assay and the ***Resistance****Plus*® MG *FleXible* assay missed some infections. Consequently, the GeneXpert had some lower performance statistics; the PPA and NPA for *M. genitalium* were 74·1% (95%CI: 65·2%-81·8%) and 97·4% (95%CI: 95·3-98·7%), respectively. The Cohen’s kappa coefficients were 0·97 for *N. gonorrhoeae* and 0·94 for *C. trachomatis*, respectively, indicating almost perfect agreement between the assays^1^. The kappa coefficient was 0·76 for *M. genitalium,* indicating substantial agreement between the assays^1^.

**Table 1. Comparison of results between GeneXpert and Hologic assays for *N. gonorrhoeae, C. trachomatis* and *M. genitalium* for patients undergoing both near-to-patient-testing and routine-testing**

| ***N. gonorrhoeae***^a^ |  | **Hologic (TMA)** | | |
| --- | --- | --- | --- | --- |
|  |  | **Detected** | **Not-detected** | **Total** |
| **GeneXpert** | **Detected** | 63 | 1 | 64 |
|  | **Not-detected** | 3 | 581 | 584 |
|  | **Total** | 66 | 582 | 648 |
| PPA = 95·5% (95%CI: 87·3-99·1%); NPA = 99·8% (95%CI: 99·0-100·0%)  Kappa statistic 0·97 (95% CI: 0·93-1·00) | | | | |
| ***C. trachomatis***^a^ |  | **Hologic (TMA)** | | |
|  |  | **Detected** | **Not-detected** | **Total** |
| **GeneXpert** | **Detected** | 110 | 6 | 116 |
|  | **Not-detected** | 6 | 530 | 536 |
|  | **Total** | 116 | 536 | 652 |
| PPA = 94·8% (95%CI: 89·1-98·1%); NPA = 98·9% (95%CI: 97·6-99·6%)  Kappa statistic 0·94 (95% CI: 0·90-0·97) | | | | |
| ***M. genitalium***^b^ |  | **Hologic (TMA)** | | |
|  |  | **Detected** | **Not-detected** | **Total** |
| **GeneXpert** | **Detected** | 86 | 11 | 97 |
|  | **Not-detected** | 30 | 406 | 436 |
|  | **Total** | 116 | 417 | 533 |
| PPA = 74·1% (95%CI: 65·2%-81·8%); NPA = 97·4% (95%CI: 95·3-98·7%)  Kappa statistic 0·76 (95% CI: 0·69-0·83) | | | | |

^a^participants eligible for inclusion were all clients tested as a contact of *N. gonorrhoeae* or *C. trachomatis,* or attending with symptoms consistent with NGU or proctitis

^b^participants eligible for inclusion were all clients tested as a contact of *M. genitalium,* attending for an MG-TOC, or attending with symptoms consistent with NGU or proctitis

*Summary*

In published studies, the Xpert® CT/NG test has been demonstrated to have consistently high sensitivities, specificities, PPA and NPA >95% across sample types when compared to two commercial assays, BD Dickinson ProbeTec ET, and Gen-Probe APTIMA Combo 2^2^, and our positive and negative agreement statistics align with these findings. For *M. genitalium* infections, which tend to have a lower organism load^3^, a number of factors could account for the different detection between assays, including sampling variability (i.e. different swabs used for ano-genital specimens in the two assays), sample dilution in larger volumes of urine or buffer (e.g. for TMA), or a difference in the limit of detection of the assays.

**References**

1. Landis JR, Koch GG. The measurement of observer agreement for categorical data. *Biometrics* 1977; **33**(1): 159-74.

2. Gaydos CA, Van Der Pol B, Jett-Goheen M, et al. Performance of the Cepheid CT/NG Xpert Rapid PCR Test for Detection of Chlamydia trachomatis and Neisseria gonorrhoeae. *J Clin Microbiol* 2013; **51**(6): 1666-72.

3. Murray GL, Danielewski J, Bodiyabadu K, et al. Analysis of Infection Loads in Mycoplasma genitalium Clinical Specimens by Use of a Commercial Diagnostic Test. *J Clin Microbiol* 2019; **57**(9).
